# Case Report: Three cases of lung large cell neuroendocrine carcinoma with clinicopathological features of SMARCA4 (BRG1) deficiency

**DOI:** 10.3389/fonc.2025.1538548

**Published:** 2025-06-30

**Authors:** Fangfang Zou, Jingdan Jia, Yizeng Wang, Xiaochun Fei, Chaofu Wang, Xiaoyan Chen

**Affiliations:** ^1^ Shandong Probincial Key Medical and Health Laboratory of Geriatric Gastrointestinal Tumor Pathology, Weihai Municipal Hospital, Cheeloo College of Medicine, Shandong University, Weihai, China; ^2^ Department of Pathology, Weihai Municipal Hospital, Cheeloo College of Medicine, Shandong University, Weihai, China; ^3^ Department of Pathology, Ruijin Hospital, Shanghai Jiao Tong University School of Medicine, Shanghai, China; ^4^ Department of Pathology, Second Affiliated Hospital of Fujian Medical University, Quanzhou, Fujian, China; ^5^ Department of Pathology, Ruijin-Hainan Hospital, Shanghai Jiao Tong University School of Medicine, Boao Research Hospital, Hainan, Qionghai, China

**Keywords:** lung neoplasms, SMARCA4-deficient, large cell neuroendocrine carcinoma, immunohistochemistry, differential diagnosis

## Abstract

**Objective:**

To investigate the clinicopathological characteristics, differential diagnosis and potential therapeutic targets of SMARCA4-deficient large cell neuroendocrine carcinoma (SD-LCNEC) in the lung.

**Methods:**

We analyzed the clinicopathological features of 3 cases of SD-LCNEC and reviewed relevant literature. Differential diagnoses were conducted using a panel of immunohistochemical antibodies.

**Results:**

The patients, aged 57 to 73 years, had tumors located in the right upper lobe, left hilum, and left upper lobe of the lung, respectively. All patients presented with lung masses. The tumors exhibited neuroendocrine carcinoma morphology, characterized by large tumor cells (nuclear diameter >3 lymphocytes), frequent mitoses, and prominent nucleoli. Tumor cells tested negative for SMARCA4 but were positive for Chromogranin-A(CgA), Synaptophysin (SYN), Insm-1, TTF-1 (partial), and Ki67 (90%). They were also negative for NapsinA, P63, P40, CK5/6, CK7, and NUT. Postoperative follow-up revealed one death, one case of Progressive Disease (PD), and one case of Stable Disease (SD).

**Conclusion:**

SD-LCNEC is a rare and clinically aggressive carcinoma, often presenting with lymph node metastasis at the initial stage. Its morphology is equivalent to LCNEC; however, immunohistochemical staining indicates the absence of SMARCA4 and a reduction in neuroendocrine markers and TTF-1. There is no standardized treatment, but SMARCA4 may serve as a potential therapeutic target. Accurate identification of the molecular subtype of SD-LCNEC is crucial.

## 1 Introduction

Large cell neuroendocrine carcinoma (LCNEC) of the lung is a rare yet highly aggressive neuroendocrine tumor, accounting for 1-3% of all primary lung cancers (1). The SWI/SNF family, a major ATP-dependent chromatin remodeling complex, encompasses the BAF and PBAF complexes. Core subunits of the BAF complex, SMARCA4 (encoding BRG1) and SMARCA2 (encoding BRM), are highly homologous, with mutations or deletions closely associated with various tumors (2), including 10% of non-small cell lung cancers. SMARCA4-deficient large cell neuroendocrine carcinoma (SD-LCNEC) is particularly rare. This study examines the clinicopathological features and differential diagnosis of three cases of lung SD-LCNEC to provide reference for diagnosis and treatment.

## 2 Materials and methods

### 2.1 Case information

Three cases of lung SD-LCNEC were diagnosed at Ruijin Hospital, Shanghai Jiao Tong University School of Medicine, from September 2021 to May 2024 were retrospectively reviewed. All slides were re-evaluated by two experienced pathologists, and the diagnoses were verified according to the WHO’s fifth edition of lung tumor classification. This was done in conjunction with patient clinical history and imaging data, along with follow-up information.

### 2.2 Methods

Specimens from three cases of SD-LCNEC were fixed in 4% neutral formalin, routinely dehydrated, embedded in paraffin, and sectioned into 4 μm thick sections for HE staining and light microscopy. Immunohistochemical analysis was performed using Roche Ventana BenchMark GX automated immunohistochemistry staining system. Primary antibodies, including CKpan, CK7, TTF-1, CgA, SYN, CD56, Insm-1, POU2F3, BRG1, BRM, NapsinA, P63, P40, Claudin4, NUT, CD117, SSTR2A, SALL4, SOX2, PD-L1, and Ki67 were procured from Fuzhou Maixin Biotechnology Development Co. PD-L1 immunohistochemistry performed using 22C3 pharmDx kit (DAKO) on Autostainer Link 48. The specific procedures adhered to the kit instructions, incorporating both positive and negative controls. BRG1 and BRM deficiency was determined by complete loss of nuclear expression in tumor cells, while background inflammatory and stromal cells exhibited positive nuclear expression. TTF-1, NUT, P63, P40, SALL4, SOX2, POU2F3, Insm-1 and Ki67 were located in the nucleus of the cells. In contrast, CgA, SYN, CK7, NapsinA, CD117 and SSTR2A were located in the cytoplasm, while Claudin4, CKpan, CD56, PD-L1(22C3) were located in the cell membrane.

### 2.3 Multiplex fluorescent PCR

Multiplex fluorescent PCR was employed to detect EGFR, EML4-ALK, ROS1, RET, NTRK1/2/3, BRAF V600E, KRAS, HER2, and MET using a gene mutation detection kit for human lung cancer.

## 3 Results

### 3.1 Clinical features

#### 3.1.1 Sex and age

Two males, one female, aged 57–73 years, with an average of 65 years and a median age of 66 years.

#### 3.1.2 Largest tumor diameter

Ranging from 2.0 to 4.5 cm, with an average of 3.0 cm.

#### 3.1.3 Smoking status

Two smokers and one non-smoker.

#### 3.1.4 Location

All tumors were located in the lung. One patient underwent a upper lobe of the left lung, which resulted in a diagnosis of poorly differentiated carcinoma and later developed neck lymph node metastasis.

#### 3.1.5 Clinical symptoms

All cases were detected during routine check-ups. One patient experienced systemic pain, while the others exhibited no obvious symptoms.

#### 3.1.6 Imaging

In case 1, PET/CT revealed an irregular mass in the apical posterior segment of the left upper lobe, along with multiple enlarged lymph nodes observed in the mediastinal aortic window and the left hilum. In case 2, PET/CT indicated metabolic abnormalities in the apical segment of the right upper lobe and the presence of soft tissue nodules; multiple metabolically enlarged lymph nodes were noted in the mediastinum (4R) and both lung hilums. In Case 3, PET/CT showed increased metabolic activity in the soft tissue of the right middle lobe, characterized by lobulation, a burr-like appearance, and pleural retraction; multiple hypermetabolic lymph nodes were observed in the mediastinum region (10L) and both lung hilums. The clinicopathological data of the three cases are summarized in **Table 1**.

**Table 1:** Clinicopathological characteristics of 3 patients with SMARCA4-deficient LCNEC.

Parameter	Case 1	Case 2	Case 3
Age (years)	73	66	57
sexes	Male	Female	Male
Location	Upper lobe of the left lung	Upper lobe of the right lung	Middle lobe of the right lung
Smoking status	NO	NO	YES
Maximum tumor diameter (cm)	2.4	4.5	2.0
Clinical manifestation	Physical examination reveals	Physical examination reveals	Physical examination reveals
Imaging		CT: right lung occupancy	CT: Patchy nodular shadows are seen in the middle lobe of the right lung near the oblique fissure.
	PET-CT: Irregular mass shadow seen in the apical posterior segment of the upper lobe of the left lung. Multiple enlarged lymph nodes in the mediastinum and left hilar.	PET-CT: Metabolic abnormalities in the apical segment of the upper lobe of the right lung increased soft tissue nodules; multiple enlarged lymph nodes in the mediastinum and both lung hilums.	PET-CT: Right lung middle lobe soft tissue with lobulation, burr and pleural pulling, and increased metabolism.
Specimen site	Left cervical lymph node puncture	Lobectomy of the upper lobe of the right lung + lymph node dissection	Pulmonary hilar lymph node dissection
Pathological diagnosis	Metastatic large cell neuroendocrine carcinoma of the lung with deletion of SMARCA4 protein expression	Complex large cell neuroendocrine carcinoma (large cell neuroendocrine carcinoma with a small amount of squamous cell carcinoma) with SMARCA4 and SMARCA2 protein expression loss	Metastatic large cell neuroendocrine carcinoma with SMARCA4 protein expression deficiency
Immunophenotype	Deletion of protein expression: BRG1	Deletion of protein expression: BRG1, BRM	Deletion of protein expression: BRG1
	Strong Expression: SYN, Insm-1	Strong Expression: SYN	Strong Expression: CD56, Insm-1
	Weak Expression: CKpan	Weak Expression: CKpan, Claudin4	Weak Expression: CKpan
	Minor expression: CK7, CgA	Minor expression: CgA, TTF-1, P40, P63	Minor expression: SYN, CgA, TTF-1
	Negative: CD56, TTF-1, NapsinA, P63, P40	Negative: CK5/6, CK7, NUT, NapsinA, CD56, Insm-1, POU2F3, CD34, SALL4, SOX2	Negative: CK7, NapsinA, CK5/6, P63, P40, SSTR2A, NUT, CD117
	Ki67 = 80%	Ki67 = 90%	Ki67 = 90%
	PD-L1 (TPS = 5%)	PD-L1 (TPS = 2%)	PD-L1 (TPS=2%)
Staging at initial visit	IIIB (cT1cN3M0)	IIIA (pT2bN2M0)	IIIB (cT1cN3M0)
Initial treatment modalities	puncture biopsy	surgical resection	Pulmonary hilar lymph node dissection
Follow-up treatment modalities	Chemotherapy + Radiotherapy	Chemotherapy + Radiotherapy	Chemotherapy + Immunotherapy
Follow-up time (months)	13	16	9
Follow-up results	Dead	Disease progression (PD)	Disease stabilization (SD)

### 3.2 Pathological histological features

**Figure 1:**
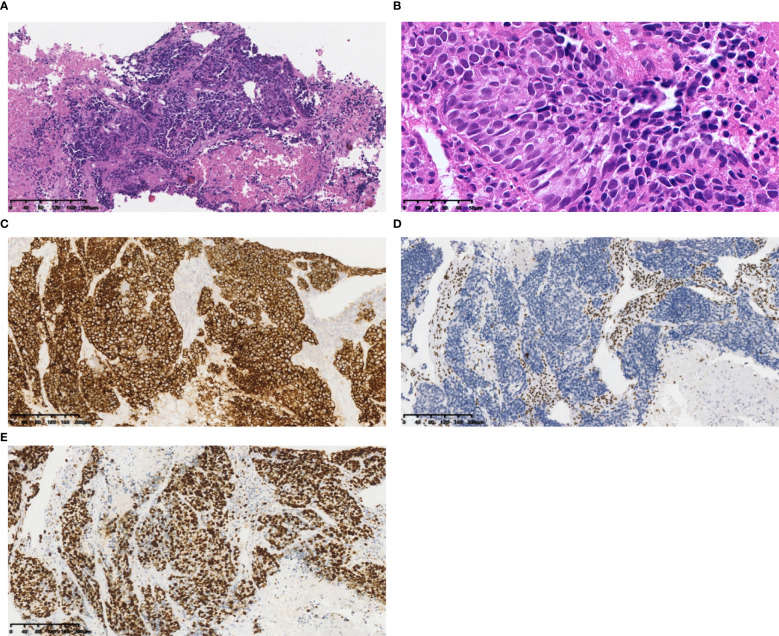
Case 1 **(A)** Tumor cells resembled small cell lung carcinoma (SCLC) with nest and beam formations, demonstrated a solid flake pattern accompanied by extensive necrosis. Hematoxylin and Eosin(HE), 10×objective, total magnification×100. **(B)** The tumor cells ranged from medium to large in size, displaying irregular nuclear contours, finely granular chromatin, and occasional nucleoli. HE 40× objective, total magnification ×400. **(C)** SYN positive. **(D)** BRG1 negative. **(E)** Ki-67 positivity rate: 80%. **(C-E)** Immunohistochemistry 10×objective, total magnification×100.

**Figure 2:**
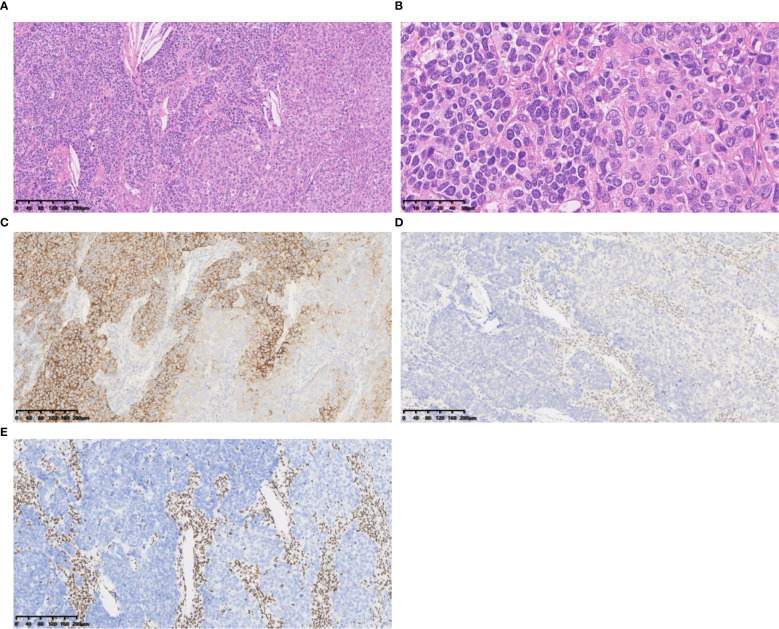
Case 2 **(A)** The tumour cells exhibited classical neuroendocrine tumour morphology, were characterized by nested, organoid, and trabecular architectures. Hematoxylin and Eosin(HE), 10×objective, total magnification×100. **(B)** The tumour cells displayed large size, abundant eosinophilic cytoplasm, coarsely granular chromatin, and prominent nucleoli. HE 40× objective, total magnification ×400. **(C)** SYN positive. **(D)** BRG1 negative. **(E)** BRM negative. **(C-E)** Immunohistochemistry 10×objective, total magnification×100.

**Figure 3:**
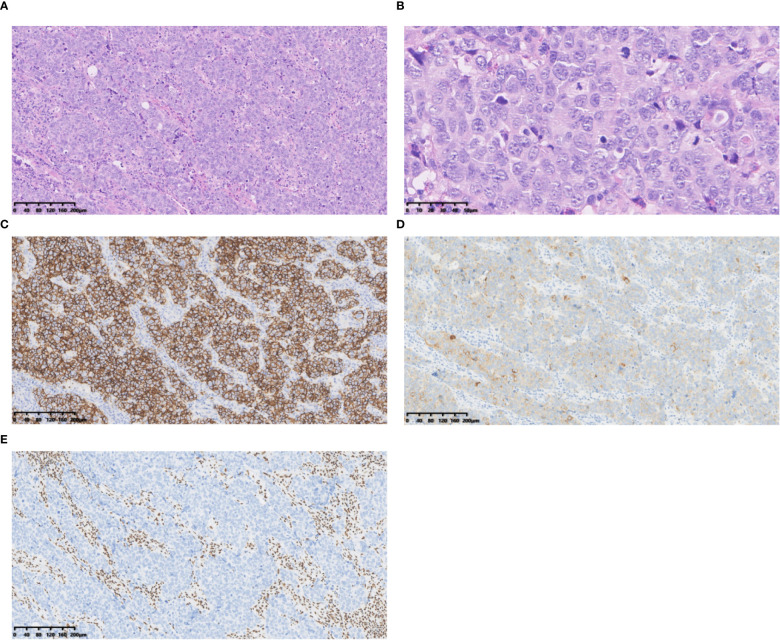
Case 3 **(A)** Tumour cells with large cytoplasmic bodies, nested clusters of trabecular pattern. Hematoxylin and Eosin(HE), 10×objective, total magnification×100. **(B)** Tumour cells with abundant cytoplasm, rough chromatin, distinct nucleoli and numerous nuclear divisions. HE 40× objective, total magnification ×400. **(C)** CD56 positive. **(D)** Syn positive. **(E)** BRG1 negative. C-E Immunohistochemistry 10×objective, total magnification×100.

SD-LCNEC may not be morphologically specific; the morphology observed in the three cases presented in this paper aligns with that of common LCNEC, displaying neuroendocrine carcinoma characteristics. Case 1, at low power (10×), resembling small cell lung carcinoma (SCLC) with nest and beam formations, demonstrated a solid flake pattern accompanied by extensive necrosis (**Figure 1A**). On high-power examination (40×), the tumor cells ranged from medium to large in size, displaying irregular nuclear contours, finely granular chromatin, and occasional nucleoli (**Figure 1B**). Cases 2 and 3, at low power (10×), exhibiting classical neuroendocrine tumor morphology, were characterized by nested, organoid, and trabecular architectures (**Figures 2A**, **3A**). On high-power examination (40×), the tumor cells displayed large size, abundant eosinophilic cytoplasm, coarsely granular chromatin, and prominent nucleoli (**Figures 2B**, **3B**). All three cases displayed large tumor cells, with nuclear diameter >3 quiescent small lymphocytes, and mitotic images >10/2mm^2^. It can be differentiated from small cell carcinoma in terms of histomorphology.

### 3.3 Immunohistochemistry

All three tumors demonstrated deficient expression of the BRG1 protein (**Figures 1D**, **2D**, **3E**), with one also exhibiting deficient expression of the BRM protein (**Figure 2E**); Additionally, all tumors expressed the neuroendocrine markers CgA, CD56 (**Figure 3C**) and SYN (**Figures 1C**, **2C**, **3D**); two tumors expressed Insm-1and TTF-1. Combining these immunohistochemical results with the neuroendocrine morphology allowed us to make the diagnosis of LCNEC. The proliferation index of Ki67 exceeded 80% (**Figures 1E**). The markers CKpan, P40, P63, Claudin4, CK5/6, CK7, NUT, and NapsinA effectively ruled out lung adenocarcinoma, squamous cell carcinoma, NUT carcinoma and Thoracic SMARCA4-deficient undifferentiated tumor (SMARCA4-UT) (see **Table 1**).

### 3.4 Multiplex fluorescent PCR

One case tested for 11 genes revealed no mutations.

### 3.5 Treatment and follow-up

In Case 1, treatment commenced with irinotecan and carboplatin for one course at the initial facility, followed by oral Anlotinib, which was discontinued after three days due to severe myelosuppression. Subsequent ultrasonography indicated cervical lymph node metastasis. After treatment with a low-dose EP regimen (etoposide and cisplatin), the cervical mass gradually increased. The patient then presented to our hospital for a lymph node biopsy, received three courses of chemotherapy with irinotecan and cisplatin, underwent particle implantation radiotherapy, and died 13 months after disease onset. In Case 2, the patient underwent right upper lobectomy with lymph node dissection and thoracoscopic pleural adhesion release. Postoperative pathological staging was IIIA (pT2bN2M0). Etoposide and carboplatin chemotherapy were administered, followed by radiotherapy targeting the focal lung areas and positive lymph node regions. A CT review 16 months post-operation revealed new foci in both lungs, with disease progression observed at 16-month follow-up (PD). In Case 3, the patient underwent pleural adhesion release and thoracoscopic mediastinal lymph node dissection, followed by immuno-EP chemotherapy (Serplulimab, cisplatin, and etoposide) after surgery. The disease remained stable (SD) at the 9-month follow-up post-surgery. The treatment and follow-up data for the three patients are shown in (**Table 1**).

## 4 Discussion

Lung SD-LCNEC, although rare, has gained attention as the SWI/SNF complex family becomes a focal point in research, as reported by Dagogo-Jack et al (3). The study of SMARCA4 deletion in a large number of lung cancer samples using second-generation sequencing technology revealed the presence of SMARCA4 deletion in neuroendocrine carcinomas, as previously reported by Gandhi et al (4). In this study, we report three cases diagnosed with SD-LCNEC at Ruijin Hospital of Shanghai Jiaotong University. All three cases presented with lymph node metastasis at the initial visit and were classified as locally advanced stage. The overall survival time of patients with advanced LCNEC was significantly shorter than that of patients with stage-matched lung adenocarcinoma (5). SD-LCNEC may not be morphologically specific compared to common LCNEC, the diagnosis is based on typical neuroendocrine morphology, neuroendocrine marker expression, and SMARCA4-deficient expression. The markers CKpan, P40, P63, Claudin4, CK5/6, CK7, NUT, and NapsinA effectively ruled out lung adenocarcinoma, squamous cell carcinoma, and NUT carcinoma. SD-LCNEC is mainly differentiated from SMARCA4-UT. According to the 2021 WHO classification of thoracic tumors, SMARCA4-UT is classified under “other tumors of epithelial origin of the lung”. It primarily affects young male smokers and is characterized by histological features such as solid sheets, islands, or nodules of cells, frequently accompanied by necrosis. The similarity of histomorphology poses challenges to diagnosis. Immunohistochemistry plays an important role in differential diagnosis. Immunohistochemistry demonstrates positivity for CD34, SOX2, and SALL4, with CKpan negativity or focal paranuclear punctate positivity, Claudin-4 negativity, and frequent double deletion of SMARCA4 and SMARCA2. While Case 2 demonstrated dual SMARCA4/SMARCA2 loss—a hallmark of SMARCA4-UT, the expression of CgA, SYN, CKpan, and Claudin4 aligned with LCNEC criteria, distinguishing it from SMARCA4-UT (typically CD34+/SOX2+/SALL4+ with epithelial marker negativity.

The literature indicates that approximately 3%-7% of lung neuroendocrine tumors harbor SMARCA2 and SMARCA4 mutations (6, 7). Notably, neuroendocrine markers and TTF-1 expression were found to decrease in the absence of SMARCA4, predominantly by SYN, but also by Insm-1, CgA or CD56 (8). In this study, all three cases exhibited weak positivity for CgA. Two cases were negative for CD56, and one case showed focal weak staining for Syn. Recent studies have demonstrated that SMARCA4 controls REST, a known suppressor of the NE phenotype, by regulating SRRM4-dependent REST transcript splicing. Moreover, inhibition of SMARCA4 activates of the ERBB family receptors ERBB2, ERBB3 and ERBB4 in SCLC, rendering these tumors sensitive to afatinib (9). Therefore, we hypothesized that this may explain the reduced expression of neuroendocrine markers observed in cases of SMARCA4 deletion.

Recent molecular genetics studies have identified three primary molecular subtypes of lung LCNEC: 1. A Small Cell Lung Cancer (SCLC)-like subtype characterized by RB1 and TP53 genomic alterations; 2. A carcinoid subtype lacking TP53 genomic mutations but exhibiting alterations in neuroendocrine tumor genes; 3. A carcinoid subtype in which SMARCA4, KRAS, FGF3/4/19, STK11, CDKN2A/B, and other genomic alterations predominate in non-small cell lung cancer-like subtypes (10–12). However, there are currently few therapeutic targets for the treatment of LCNEC molecular subtypes, and so far only everolimus (an mTOR inhibitor) has been identified as a targeted drug (1). In 1 of our 3 cases, no driver mutations were detected through 11 genetic tests related to lung cancer treatment. Emerging evidence highlights the role of SMARCA4 in lung cancer maintenance, carcinogenesis, and treatment (13). Inactivation of the SMARCA4 gene, which encodes BRG1 protein, increases tumor invasiveness and affects sensitivity to conventional platinum-based chemotherapeutic agents but may increase tumor sensitivity to EZH2 inhibitors, CDK4/6 inhibitors (3). All our 3 cases were treated with platinum-based chemotherapy and additional therapies, resulting in one death and one case of disease progression. In case 1, the patient received a low-dose EP chemotherapy regimen combined with particle implantation radiotherapy. Unfortunately, the patient succumbed to the disease 13 months after diagnosis. I case 2, the patient underwent surgical resection followed by adjuvant platinum-based chemotherapy and radiotherapy targeting the lung and lymph node regions. Despite multimodal treatment, disease progression was observed during the 16-month follow-up, with the emergence of new pulmonary lesions. Several small retrospective studies (1, 14, 15) suggest that immunotherapy demonstrates greater efficacy compared to drug toxicity in patients with LCNEC. Additionally, positive expression of PD-L1 (TPS ≥1%) was associated with SMARCA4 deficiency (16), which may predict response to immunotherapy. This is supported by Case 3. In case 3, the patient received platinum-based chemotherapy and immunotherapy, and at the 9-month postoperative follow-up, the disease remained stable (SD). As studies increasingly correlate SMARCA4 with lung cancer treatment and prognosis, the accurate identification of SMARCA4-deficient tumor subtype becomes critical and may necessitate specific therapeutic strategies. we will expect to explore and develop novel targeted therapies for SMARCA4-deficient tumors, including CDK4/6 inhibitors and EZH2 inhibitors. Further clinical trials are critical to evaluate targeted therapies and optimize therapeutic option for this rare subtype.

## 5 Summary

In this study, we retrospectively analyzed the clinicopathological data of three cases of SMARCA4-deficient large cell neuroendocrine carcinoma (SD-LCNEC), reporting for the first time the distinct features of this rare and aggressive subtype characterized by early metastasis and reduced expression of neuroendocrine markers. Our findings emphasize the critical role of SMARCA4 immunohistochemical analysis in accurately identifying SD-LCNEC, particularly in cases with low neuroendocrine marker and TTF-1 expression. Moving forward, expanding clinical case collections and exploring targeted therapies, such as CDK4/6 and EZH2 inhibitors, will be essential to improve patient outcomes and advance our understanding of SD-LCNEC’s biological behavior, classification, and treatment strategies.

## Data Availability

The original contributions presented in the study are included in the article/Supplementary Material. Further inquiries can be directed to the corresponding author.
